# Dose-dependent toxicity profile and genotoxicity mechanism of lithium carbonate

**DOI:** 10.1038/s41598-022-17838-0

**Published:** 2022-08-05

**Authors:** Selin Sipahi Kuloğlu, Emine Yalçin, Kültiğin Çavuşoğlu, Ali Acar

**Affiliations:** 1 Institute of Science, Giresun University, Giresun, Turkey; 2 Department of Biology, Faculty of Science and Art, Giresun University, Giresun, Turkey; 3 Department of Medical Services and Techniques, Vocational School of Health Services, Giresun University, Giresun, Turkey

**Keywords:** Genetics, Cell division, Chromosomes

## Abstract

The increasing widespread use of lithium, which is preferred as an energy source in batteries produced for electric vehicles and in many electronic vehicles such as computers and mobile phones, has made it an important environmental pollutant. In this study, the toxicity profile of lithium carbonate (Li_2_CO_3_) was investigated with the *Allium* test, which is a bio-indicator test. Dose-related toxic effects were investigated using Li_2_CO_3_ at doses of 25 mg/L, 50 mg/L, and 100 mg/L. The toxicity profile was determined by examining physiological, cytotoxic, genotoxic, biochemical and anatomical effects. Physiological effects of Li_2_CO_3_ were determined by root length, injury rate, germination percentage and weight gain while cytotoxic effects were determined by mitotic index (MI) ratio and genotoxic effects were determined by micronucleus (MN) and chromosomal aberrations (CAs). The effect of Li_2_CO_3_ on antioxidant and oxidant dynamics was determined by examining glutathione (GSH), malondialdehyde (MDA), catalase (CAT) and superoxide dismutase (SOD) levels, and anatomical changes were investigated in the sections of root meristematic tissues. As a result, Li_2_CO_3_ exhibited a dose-dependent regression in germination-related parameters. This regression is directly related to the MI and 100 mg/L Li_2_CO_3_ reduced MI by 38% compared to the control group. MN and CAs were observed at high rates in the groups treated with Li_2_CO_3_. Fragments were found with the highest rate among CAs. Other damages were bridge, unequal distribution of chromatin, sticky chromosome, vagrant chromosome, irregular mitosis, reverse polarization and multipolar anaphase. The genotoxic effects were associated with Li_2_CO_3_-DNA interactions determined by molecular docking. The toxic effects of Li_2_CO_3_ are directly related to the deterioration of the antioxidant/oxidant balance in the cells. While MDA, an indicator of lipid peroxidation, increased by 59.1% in the group administered 100 mg/L Li_2_CO_3_, GSH, which has an important role in cell defense, decreased by 60.8%. Significant changes were also detected in the activities of SOD and CAT, two important enzymes in antioxidant defense, compared to the control. These toxic effects, which developed in the cells belonging to the lithium-treated groups, were also reflected in the tissue anatomy, and anatomical changes such as epidermis cell damage, cortex cell damage, flattened cell nucleus, thickening of the cortex cell wall and unclear vascular tissue were observed in the anatomical sections. The frequency of these changes also increased depending on the Li_2_CO_3_ dose. As a result, Li_2_CO_3_, which is one of the lithium compounds, and has become an important contaminant in the environment with increasing technological developments, caused a combined and versatile toxicity in *Allium cepa* L. meristematic cells, especially by causing deterioration in antioxidant/oxidant dynamics.

## Introduction

Industrialization and advances in technology have not only made people's lives easier, but also have caused various problems to arise. With the technological developments, the use of electronic products has increased day by day, and the rapid production and consumption of these products has brought many problems. Electronic waste, called e-waste is unused electrical and electronic devices. E-waste contains many materials such as plastic, metal and glass, and when they degrade, dangerous substances are released into the environment. E-waste contains more than a thousand substances in its structure and includes metals such as lead, mercury, cadmium, chromium and lithium^1,2^. Contamination of these metals to the environment adversely affects both environmental safety and all organisms. Lithium, which is used as an energy source in electric vehicles batteries and many electronic vehicles such as computers and mobile phones, is an important pollutant that contaminates the environment through e-waste. On-site disposal of lithium-hydride and lithium-deuterium material, waste from the electronics, fabric, ceramics and cosmetics industries are the main sources of environmental lithium contamination^3^. Lithium, which is used for industrial purposes in colorants, batteries and metal alloys, contaminates water resources and soil and causes serious pollution^4^.

It has been reported that lithium compounds, which are widely distributed in nature, are found at rates of 30 mg/kg in the earth's crust, 25 mg/kg in the soil, 2 mg/kg in drinking water, 170–190 mg/L in sea water and 2 ng/m^3^ in the atmosphere^3^. Due to its wide distribution, lithium is easily taken up by plants and reaches other organisms through the food chain. Lithium uptake and accumulation differ among plant species. Some plants are hyper- or bio- accumulators of lithium, while others keep their lithium intake below the threshold level^5^. Some metals are toxic to plants, and increasing their concentration from the optimum level delays plant growth and yield. Although the toxic effects of lithium on plants are not yet clear, it is reported that lithium salts are highly toxic and cause a significant reduction in plant growth by triggering the formation of necrotic zones. However, different plant species exhibit different behaviors in terms of sensitivity and tolerance to lithium toxicity. Although studies have reported that low concentrations of lithium can stimulate growth in some plant species, it is reported that high concentrations of lithium reduce or completely inhibit growth. It has been suggested that some harmful effects of lithium, whose toxicity mechanism is still unclear, may be related to oxidative stress^3–5^. It is reported that the production of free radicals and oxidative stress occur in severe lithium exposure. Lithium induces the formation of free oxygen radicals with Fenton-type reactions and can cause oxidative damage in the cell. Oxidative stress formation under lithium stress also reduces plant growth^6^. In the presence of oxidative stress, macromolecules such as DNA, lipid and protein are damaged, and the physiological and biochemical pathways in the cell are disrupted. Free radicals mainly attack the unsaturated lipids acids of cell membranes, causing lipid peroxidation and cell membrane damage. Proteins can also be oxidized as a result of oxidative stress, and amino acids in the protein structure are converted into various intermediates by oxidation. These changes in protein structure cause loss of function. It is also known that DNA damages occur in the presence of oxidative stress in cells. Oxidative damage, especially in purine bases, causes abnormalities in DNA^7,8^. Oxidative stress induced by lithium can cause various damages to cellular macromolecules and may also cause regression in plant growth. Some of the other potential effects of high concentrations of lithium are decreased chlorophyll content and photosynthesis, DNA condensation, inhibition of protein and amide biosynthesis, and conformational changes in DNA^9^. In this study, the toxic effects of lithium on meristematic cells, whose toxicity mechanism and possible effects in higher plants have not yet been revealed in detail, were investigated. This toxicity risk monitoring study was performed using the *Allium* test. Higher plants such as *A. cepa* are important indicator organisms used to investigate the cytotoxic and mutagenic effects of chemical agents. Compared to other tests, the *Allium* test is an easy to apply, fast and cost-effective method. The results of *Allium* test show important compatibility with other toxicity tests. Cytotoxicity tests with human lymphocyte cells and algal cells show high compatibility with the results of the *Allium* test^10^. In this context, the *Allium* test is the first alternative test system in determining the possible multifaceted toxicity caused by environmental toxic agents. *A. cepa* has an oxidase-enzyme system that exhibits similar activity to detoxifying mechanisms in mammals. This similarity provides a high correlation between the *Allium* test and the toxicity tests performed in mammals. With the *Allium* test, substances that cause toxic effects in eukaryotes can be detected and the results obtained can be used as a preliminary assessment in all animal and plant biodiversity. The *Allium* test allows the investigation of not only the cytological or genetic effects of various natural or synthetic components, but also the physiological, clastogenic, aneugenic, anatomical and biochemical effects^11,12^.

In this study, physiological, cytotoxic, genotoxic, biochemical and anatomical effects of lithium carbonate (Li_2_CO_3_) compound on meristematic cells were investigated using the *Allium* test. Different analysis methods were used within the scope of the study. In this way, it is aimed to determine the multiple toxicity of lithium. The data obtained in each analysis were correlated with each other and the mechanism of toxicity was tried to be clarified. The potential of lithium to induce oxidative stress was evaluated by investigating changes in the antioxidant/oxidant balance. For this purpose, glutathione (GSH) and malondialdehyde (MDA) levels, catalase (CAT) and superoxide dismutase (SOD) activities were measured in meristematic cells. Mitotic index (MI) rates were used to determine the cytotoxic effects and the micronucleus (MN) and chromosomal aberrations (CAs) frequencies were investigated to determine the genotoxic effects. In particular, the effect of Li_2_CO_3_ on DNA fragmentation was investigated in order to evaluate its genotoxic mechanism of action, and the interaction between Li_2_CO_3_-DNA was investigated by molecular docking. The possible physiological effects of Li_2_CO_3_ were investigated using closely related parameters such as germination rate, root length, weight gain and relative injury rate analyses. Anatomical changes as a result of Li_2_CO_3_ exposure were also determined by root tip sections. The potential toxicity mechanism was interpreted by correlating all the obtained data with each other.

## Material and methods

### Test material and chemical

*A. cepa* bulbs (2n = 16) were purchased from a commercial market in Giresun (Turkiye), and Li_2_CO_3_ (CAS No: 554-13-2), carmine (CAS No: 1390-65-4), low melting agarose (CAS No: 39346-81-1), ethidium bromide (CAS No: 1239-45-8) were purchased from Merck and Sigma-Aldrich.

### Experimental process

*Allium* test was used to examine Li_2_CO_3_ toxicity and for this purpose bulbs were divided into four groups as Control (Group I), 25 mg/L Li_2_CO_3_ (Group II), 50 mg/L Li_2_CO_3_ (Group III), 100 mg/L Li_2_CO_3_ (Group IV).

**Figure 1:**
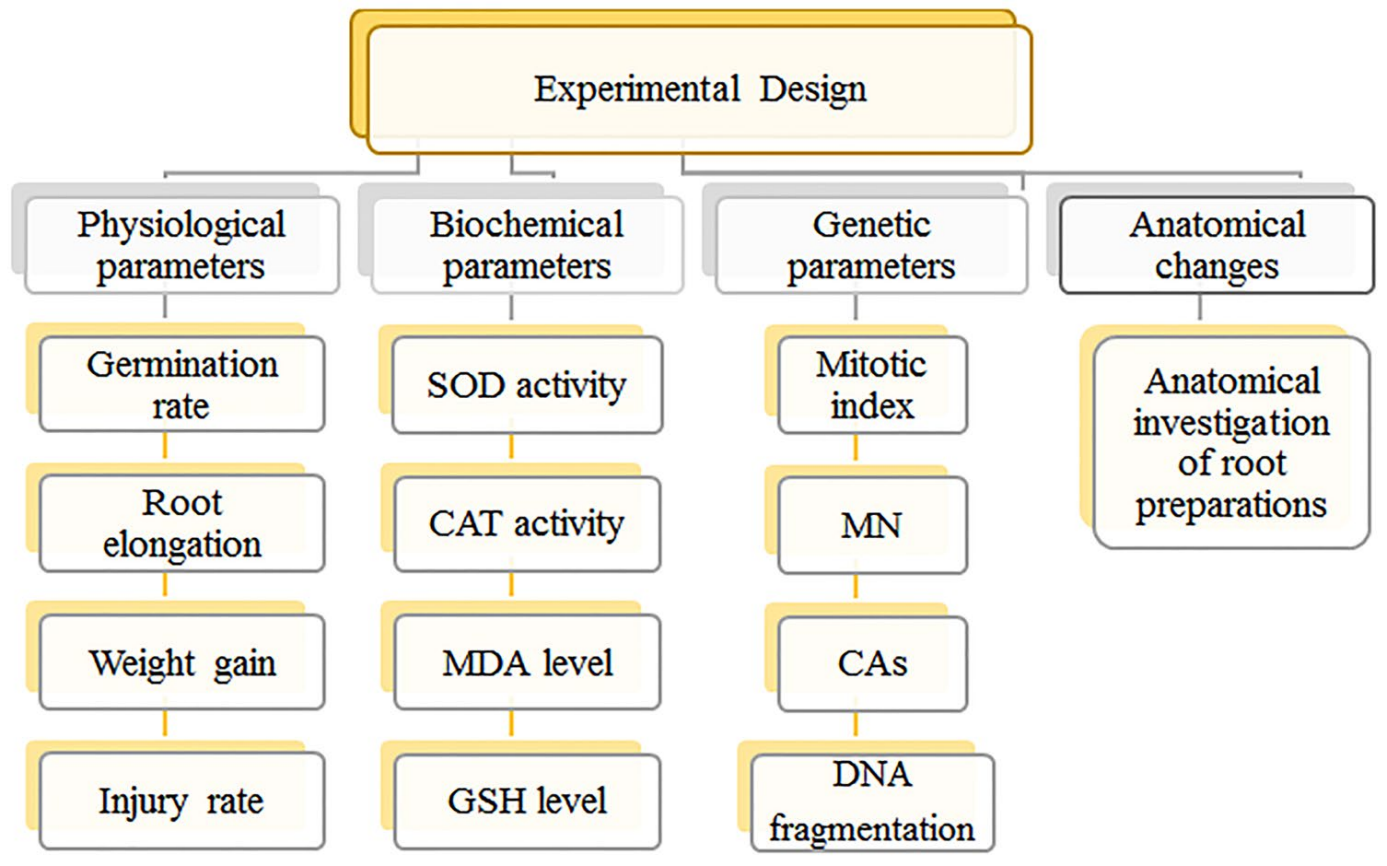
Experimental design of the study.

A preliminary study was conducted to determine the EC_50_ value, based on the dose ranges known to inhibit growth in various plants in the literature^13^. The EC_50_ value was investigated in dose ranges of 0–120 mg/L and was determined as 50 mg/L. Three different doses were used in the study, EC_50_ value (50 mg/L), half (25 mg/L) and double (100 mg/L) of this value. The bulbs in the control group were germinated with tap water, and the bulbs in the treatment groups were germinated with three different doses of Li_2_CO_3_. The germination process was continued at 24 °C for 72 h without interruption. The beakers were checked every twenty-four h and the decreasing solution were added. At the end of the period, the germinated root tips were washed with distilled water, cut into approximately 1 cm length, and prepared for spectrophotometric measurements and microscopic observations by applying routine homogenization and crushing preparation processes^14^. Toxicity profile was obtained by using physiological, cytogenetic, biochemical and anatomical parameters in root tips obtained from bulbs germinating in solutions containing Li_2_CO_3_, and all parameters tested in the study are given in Fig. 1.

### Physiological parameters

The effects of Li_2_CO_3_ doses on root elongation were determined by measuring with a millimetric ruler of the radicle length, which is the structure in the plant embryo and forming the root. The effects on the weight gain were determined by weighing the bulb weights before and after the application with the help of precision scales. Relative injury rate and the effects on germination rate (GR) were determined with the help of Eqs. (1) and (2), respectively^12^.

1 $$ {\text{Germination\;rate}}\;\left( \% \right) = \left[ {{\text{Number}}_{{{\text{germinated\;bulb}}}} /{\text{ Number}}_{{{\text{total\;bulb}}}} } \right] \times {1}00 $$

2 $$ {\text{Relative\;injury\;rate}} = \left[ {\% {\text{GR\;in\;control}} - \% {\text{GR\;in\;each\;group}}} \right]/\left[ {\% {\text{GR\;in\;control}}} \right] $$

### Cytotoxic and genotoxic effects

Cytotoxic effects of Li_2_CO_3_ were determined by MI rates and genotoxic effects were investigated with MN and CAs frequencies in meristematic cells. Acetocarmine crushing technique was used for the detection of CAs and MN. Root tips were cut about 1 cm long, fixed in “Clarke” solution for 2 h, washed in ethyl alcohol (96%) for 15 min, hydrolyzed in 1 N HCl (at 60 °C) for 17 min, and kept in glacial acetic acid (45%) for 30 min. In the last stage, root tips were stained with acetocarmine for 24 h, placed on a slide and crushed with a coverslip. CAs and MN observations were performed under the Irmeco IM-450 TI model research microscope and photographed at × 500 magnification^15^. Three criteria suggested by Fenech et al.^16^ were taken into account in the detection of MN. MI, which shows the ratio of cells undergoing mitosis in a cell population, was determined with the help of Eq. (3).

3 $$ {\text{MI\;rate}}\;\left( \% \right) = \left[ {{\text{number\;of\;divided\;cells}}/{\text{total\;number\;of\;cells}}} \right] { \times }{1}00 $$

### Comet assay

The method of Chakraborty et al.^17^ was followed for comet assay. The procedures were carried out in low light to minimize DNA degradation and examined using a fluorescence microscope. Comets were evaluated using Comet Assay software (CASP) version 1.2.3b using tail DNA length parameters^18^. A total of 1.000 cells were examined for DNA damage in each group, with 100 cells examined in each bulb. The degree of DNA damage was graded on a scale of 0 to 4 based on the severity of DNA damage. The cells were divided into five groups depending on the length of their tail DNA, which ranged from zero to four.

The total DNA damage per group, expressed as arbitrary units, was calculated using Eq. (4).

4 $$ Arbitrary\;unit = \mathop \sum \limits_{i = 0}^{4} Nix i $$

*i* degree of damage (0, 1, 2, 3, 4), *Ni* the number of cells in *i* degree.

### Antioxidant/oxidant dynamics

The effects of Li_2_CO_3_ on antioxidant/oxidant balance were investigated with GSH, MDA, SOD and CAT levels in root meristematic cells. Root MDA levels were measured by applying the method suggested by Unyayar et al.^19^ and the MDA levels are shown as µM/g FW. GSH levels were analyzed by sulfhydryl level measurement as described by Kurt et al.^20^ and expressed as µmol/g. For enzyme activity measurements, enzyme extraction procedure was carried out at + 4 °C. SOD and CAT activities were measured using the method proposed by Çavuşoğlu et al.^21^. SOD activity and CAT activities were shown as U/mg FW and OD_240nm_min/g, respectively. MDA, GSH, SOD and CAT analysis were performed in triplicate.

### Anatomical alterations

Root tips were cut 1 cm long, washed with distilled water, placed between styrofoam material and cross-sectioned with a sterile razor blade. Sections were placed on a slide, stained with methylene blue (5%) for 2 min and covered with a coverslip. Anatomical observations were made under a research microscope and photographed at × 200 magnification^22^.

### Molecular docking

Molecular docking analysis was used for interactions of Li_2_CO_3_ with different DNA molecules. The 3D structures of, B-DNA dodecamer (PDB ID: 195d)^23^, DNA (PDB ID: 1cp8)^24^ and B-DNA dodecamer (PDB ID: 1bna)^25^ molecules were obtained from the protein data bank. The 3D structure of Li_2_CO_3_ (DB Accesion Number: DB14509) was retrieved from the Drugbank. Energy minimization of DNA molecules was done with Gromos 43B1 using Swiss-PdbViewer^26^ (v.4.1.0) software whereas energy minimization of the 3D structure of Li_2_CO_3_ was accomplished with the Universal Force Field (UFF) employing Open Babel v.2.4.0 software^27^. The molecular docking process was carried out with the grid box containing the entire structure of DNA molecules. Since the atomic parameters and charge of the lithium element are not available by default in the AutoDock software, the parameters of the lithium atom have been added to the AD4_parameters.dat file. The docking and 3D visualizations were obtained with Biovia Discovery Studio 2020 Client.

### Statistical analysis

Statistical analyzes were performed by using SPSS Statistics 22 (IBM SPSS, Turkey) program. All data are shown as mean ± standard deviation (SD). The statistical significance between the means was determined with the help of one-way analysis of variance, “One-way ANOVA” and “Duncan” tests. Obtained values were considered statistically significant when p < 0.05.

## Results and discussion

### Li_2_CO_3_ effects on germination related parameters

The effects of Li_2_CO_3_ application on some physiological parameters are given in Table 1. In the control group, root length, germination rate and weight gain were 4.20 ± 0.95 cm, 98% and 5.16 g, respectively. Important decreases in physiological parameters were detected in Li_2_CO_3_ applied groups compared to the control. As the Li_2_CO_3_ dose increased, the decrease in all parameters also enhanced. 100 mg/L Li_2_CO_3_ application decreased germination rate, root length and weight gain by 39.7%, 54.7% and 27%, respectively. The relative injury rate was calculated in the groups and the highest injury rate was 0.39 in Group IV, which was administered 100 mg/L Li_2_CO_3._ These results showed that Li_2_CO_3_ caused physiological damages in *A. cepa.* These changes observed in physiological parameters in *A. cepa* can be explained by the inhibitory properties of Li_2_CO_3_ on photosynthesis reactions. Lithium reduces photosynthetic activity by damaging the chloroplast ultrastructure, inhibiting electron chain reactions, or replacing with Mg in chlorophyll^3^. Inhibition of photosynthetic reactions in plants causes a decrease in weight gain, germination percentage and root elongation, resulting in a slowdown in physiological development. Root elongation and weight gain also occur in plants with germination. Delays in germination cause disruptions in other physiological processes. The decrease in all three parameters tested in the lithium-administered groups confirms this hypothesis. There are similar studies in the literature that support our findings. Kalinowska et al.^28^ stated that increasing lithium doses in lettuce plant decreased root and shoot biomass. Hawrylak-Nowak et al.^9^ determined that exposure to 25 mg/dm^3^ lithium in sunflower caused a 16% decrease in carotenoid content, and the appearance of necrotic spots on leaves in corn, a decrease of approximately 45% in chlorophyll a and b content and a 67% decrease in carotenoid content. Gayathri et al.^29^ reported that the germination rate of *A. cepa* decreased depending on the lithium concentration dose and determined a germination rate of 73% at 50 ppm, 57% at 75 ppm and 41% at 100 ppm.

**Table 1:** Li_2_CO_3_ toxicity on physiological parameters of *A. cepa*.

Groups	Germination rate (%)	Root length (cm)	Initial weight (g)	Final weight (g)	Weight gain (g)	Relative injury rate
Group I	98	4.20 ± 0.95^a^	8.50 ± 1.40	13.66 ± 1.79	5.16^a^	0.00
Group II	85	3.50 ± 0.87^b^	8.69 ± 1.43	12.69 ± 1.70	4.00^b^	0.13
Group III	74	2.70 ± 0.63^c^	8.42 ± 1.36	11.27 ± 1.64	2.85^c^	0.24
Group VI	59	1.90 ± 0.56^d^	8.47 ± 1.38	9.97 ± 1.42	1.50^d^	0.39

50 bulbs were used for germination percentage and 10 bulbs were used for root length and weight gain. Data with different letters^(a–d)^ in the same column are statically significant at *P* < 0.05.

### Li_2_CO_3_ effects on antioxidant/oxidant dynamics

**Figure 2:**
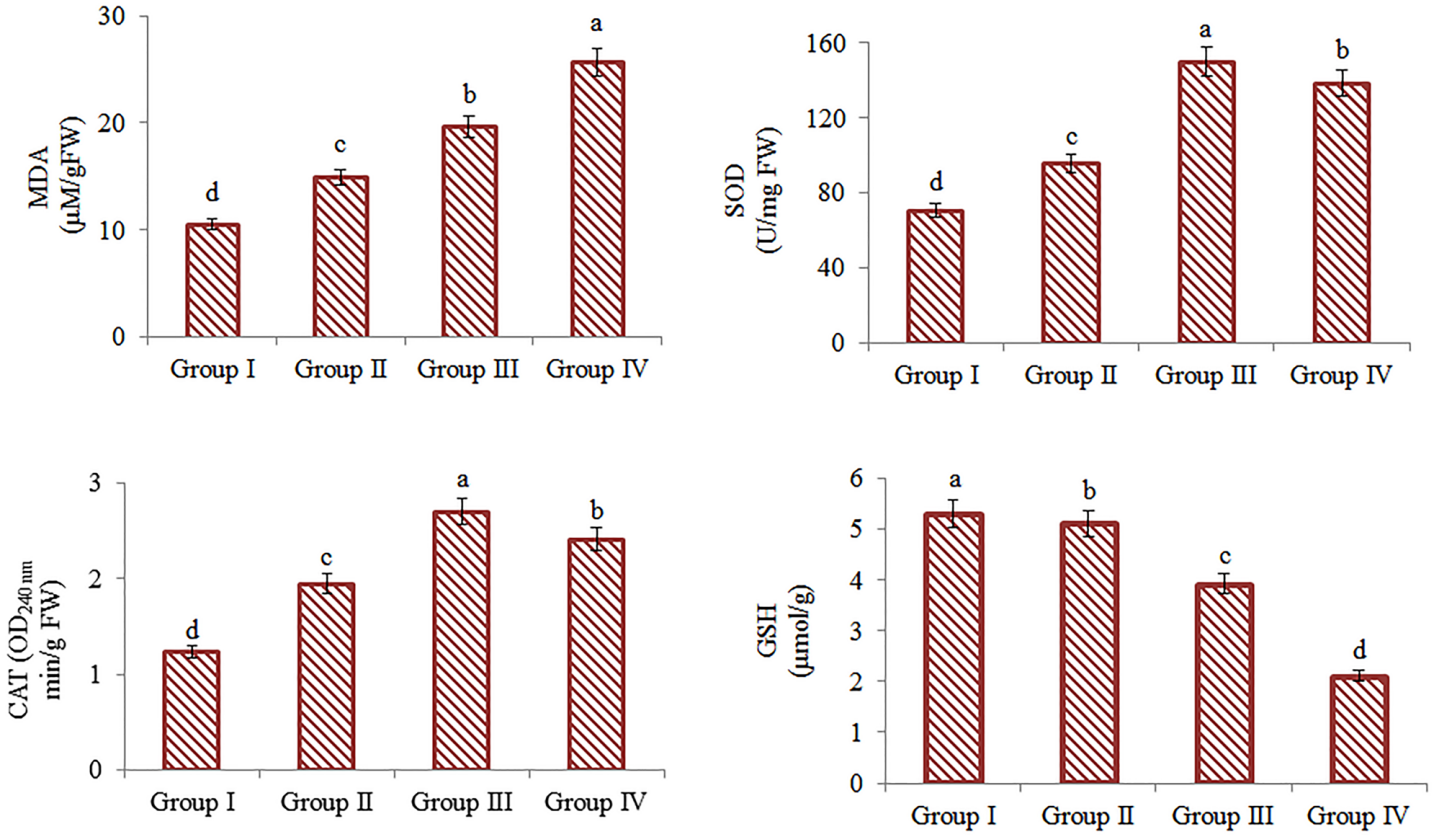
Toxic effects of Li_2_CO_3_ on antioxidant/oxidant parameters of *A. cepa*. Data with different letters^(a-d)^ in the columns are statically significant at *P* < 0.05. All analysis was performed in triplicate.

The effects of Li_2_CO_3_ application on antioxidant/oxidant balance in *A. cepa* are given in Fig. 2. To determine this effect, GSH and MDA levels, CAT and SOD activities were examined. 25, 50 and 100 mg/L Li_2_CO_3_ administration increased MDA levels by 29.5%, 46.4% and 59.1%, respectively, compared to the control. In the groups treated with Li_2_CO_3_, a decrease in GSH level was observed and the most significant decrease was 60.8% in the group treated with 100 mg/L Li_2_CO_3_. The severity of all these changes in MDA and GSH levels increased depending on the dose, and accordingly, the antioxidant/oxidant balance was impaired. This effect of Li_2_CO_3_ application is closely related to oxidative stress induction in cells. Lithium provides the emergence of free radicals from Fenton-type reactions and causes oxidative damage^3^. It has also been reported that lithium reduces chlorophyll content and photosynthesis, which is due to increased free radical production. Lithium toxicity and excessive free radical production cause oxidative stress in plants^9^. As a result of lipid peroxidation, toxic and mutagenic intermediates such as MDA are formed, cell membrane structure is deteriorated and cell integrity is damaged^30^. The endogenous antioxidant defense system of the cell provides protection against free radicals formed in the cell. GSH has an important place in the endogenous defense system and is widely found in plant organelles such as the endoplasmic reticulum, cytosol, mitochondria, chloroplast, vacuole and peroxisome. Decreased GSH level and increased MDA level in cells causes increased oxidation and disruption of antioxidant/oxidant balance^19^. It has been reported in many studies in the literature that lithium compounds cause the antioxidant/oxidant balance to deteriorate. Hawrylak-Nowak et al.^9^ reported that the lipid peroxidation level and MDA production in sunflower and corn plants increased in the presence of 50 mg/dm^3^ of lithium, and this situation disrupted the membrane integrity. Another evidence Li_2_CO_3_ administration disrupts the antioxidant/oxidant balance is the changes in SOD and CAT activities. Li_2_CO_3_ administration caused significant changes in enzyme activities compared to the control. 25 mg/L and 50 mg/L Li_2_CO_3_ treatment caused an increase in activities by inducing SOD and CAT enzymes. 50 mg/L Li_2_CO_3_ increased SOD activity by 53% and CAT activity by 54% compared to the control. Administration of 100 mg/L Li_2_CO_3_, the highest dose used in this study, caused a regression in both enzyme levels, but despite this regression, the enzyme levels remained above the control levels. Plants have different mechanisms to cope with oxidative stress and induction of antioxidant enzyme activities such as CAT and SOD is one of these mechanisms. SOD and CAT enzymes are two enzymes that play an important role in the scavenging free radicals. It has been reported by many studies that these two enzymes are induced in the presence of oxidative stress. Nciri et al.^31^ found that 1 mM lithium application increased SOD activity while decreasing glutathione peroxidase (GPx) activity In another study, it was reported that lithium application increased SOD, CAT, ascorbate peroxidase (APx) enzyme activities in spinach shoots^32^. While antioxidant enzyme activities are induced to cope with oxidative stress in cells, excessive stress can also cause denaturation of these enzymes. In this study, the decrease in SOD and CAT activities in the group administered 100 mg/L Li_2_CO_3_ as the highest dose can be explained by potential denaturation. Oxidative stress induced by free radicals triggers deterioration in the structure of proteins, oxidation of amino acids and formation of carbonyl groups. Such changes in protein structure cause loss of function and disruptions in various biochemical processes in the cell^33^. The decrease in SOD and CAT activities can be explained by these effects of oxidative stress. Similarly, it is reported in the literature that high-dose lithium applications inhibit antioxidant enzymes^30^.

### Cytotoxic effects of Li_2_CO_3_

**Figure 3:**
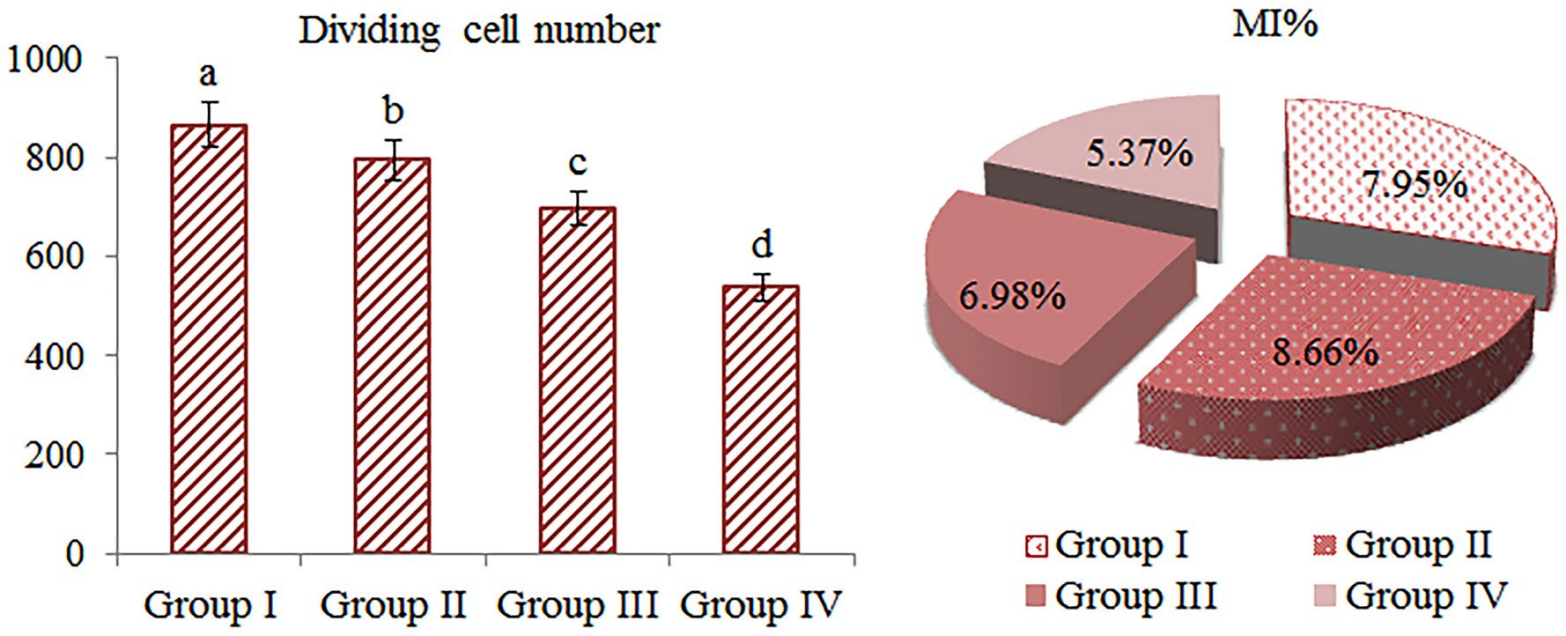
Effects of Li_2_CO_3_ application on dividing cell number and MI in meristematic cells. The MI rate was calculated by analyzing 10.000 cells in each group. Data with different letters^(a-d)^ in the columns are statically significant at *P* < 0.05.

Cytotoxic effects Li_2_CO_3_ application on *A. cepa* bulbs were investigated by MI analyses. The effects of Li_2_CO_3_ on MI in *A. cepa* are given in Fig. 3. While 866 cells were divided within 10.000 cells in the control, this number decreased to 537 in the 100 mg/L Li_2_CO_3_ administered group. Briefly, Li_2_CO_3_ application triggered a remarkable decrease in MI compared to the control. MI is a parameter used to determine the cytotoxicity of chemicals as an indicator of cell proliferation^34^. 25, 50 and 100 mg/L Li_2_CO_3_ administration reduced MI rates by 8.2%, 19.4%, and 38%, respectively, compared to the control. Decreased MI rates compared to control are indicative of slower mitotic cell divisions in the meristem cells. In addition, the decrease in MI is consistent with the reduction of root elongation, germination percentage and weight gain, which are associated with growth parameters. In similar studies it was reported that different metal ions have a regressive effect on MI. Yalçın et al.^35^ reported that HgCl_2_ application reduced MI in *A. cepa* bulbs. Likewise, Macar et al.^36^ determined that Co(NO_3_)_2_ stress reduced MI and showed a cytotoxic effect in *A. cepa*. Kikuda et al.^37^ reported significant changes in MI rates of *A. cepa* germinated with lithium-containing Buritis Lake water.

### Genotoxic effects of Li_2_CO_3_

**Figure 4:**
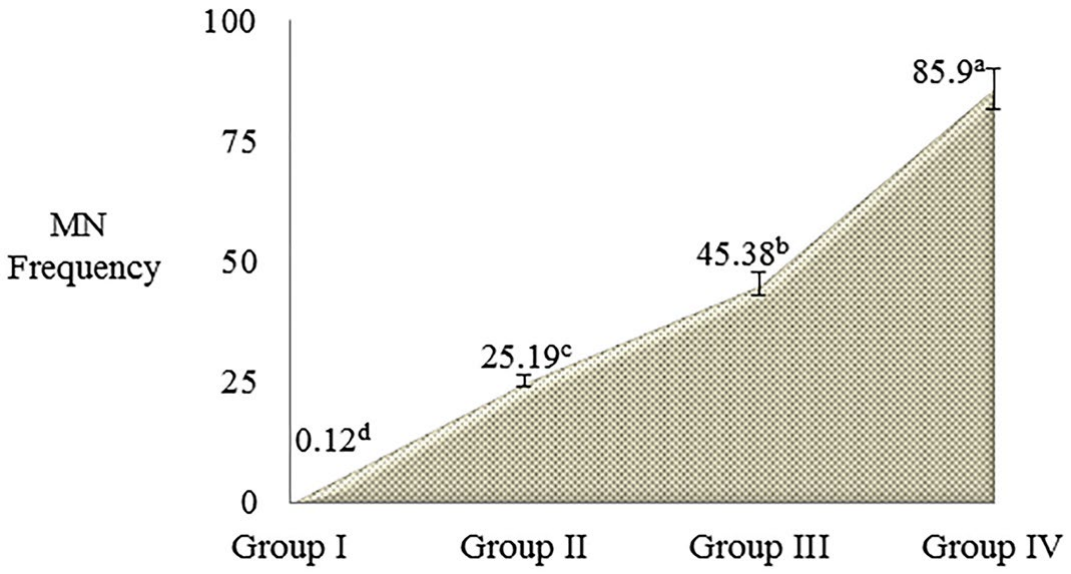
Effects of Li_2_CO_3_ application on MN frequency in meristematic cells. Data are shown as mean ± SD (n = 10). MN numbers were calculated by analyzing 1.000 cells in each group. Data with different letters^(a-d)^ in the same column are statically significant at *P* < 0.05.

**Figure 5:**
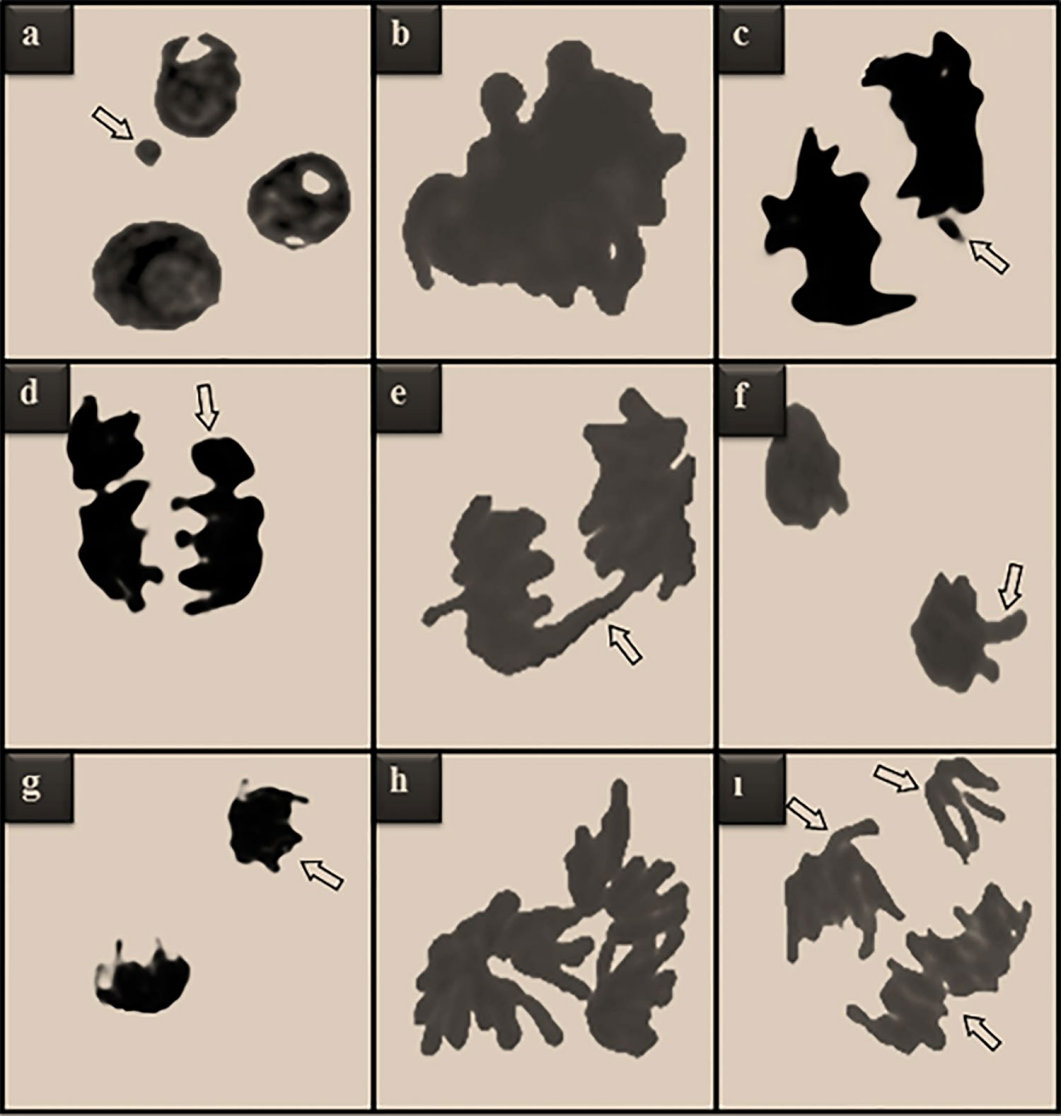
CAs induced by Li_2_CO_3_. MN in interphase (**a**), sticky chromosome in prophase (**b**), fragment in anaphase (**c**), unequal distribution of chromatin in anaphase (**d**), bridge in anaphase (**e**), vagrant chromosome in telophase (**f**), reverse polarization in telophase (**g**), irregular mitosis in metaphase (**h**), multipolar anaphase (**ı**).

The effects of Li_2_CO_3_ on CAs and MN frequencies, which indicate genotoxic effects, are given in Table 2 and Figs. 4,  5. While a few statistically insignificant MN, sticky chromosome and unequal distribution of chromatin were found in the control group, the frequency of abnormalities increased depending on the dose of Li_2_CO_3_. Different types of aberrations such as bridge, sticky chromosome, fragment, unequal distribution of chromatin, vagrant chromosome, reverse polarization, irregular mitosis and multipolar anaphase were detected in Li_2_CO_3_ applied groups. Essentially, all CAs types represent aberrant mitotic divisions. Toxic effects induced by Li_2_CO_3_ exposure occur through many mechanisms. One of the mechanisms of action of lithium stress is disruptions in nucleic acid metabolism. High concentrations of lithium can inhibit biosynthesis of protein, DNA condensation, produce toxic effects on nucleic acid metabolism or induce conformational change in DNA^3^. It has been reported in the literature that many metals such as Li_2_CO_3_ reach the environment and organisms and cause various CA formations. Sticky chromosome, highly induced by lithium application, is the result of increased chromosomal condensation, de-polymerization of DNA and partial dissolution of nucleoproteins. Sticky chromosomes, often irreversible and possibly leading to cell death, indicate highly toxic effects. Fragment, another type of CA detected at high frequency as a result of lithium application, shows that it causes breaks in DNA. These fragments transform into MN at later stages of cell division^38^. In parallel with our study, Yalçın et al.^35^ reported that HgCl_2_ caused an increase in MN and various types of CAs in *A. cepa*. Similarly, Macar et al.^36^ reported that Co stress showed a remarkable increase in terms of CAs and MN in *A. cepa.* Liu et al.^39^ showed that different metal ions can cause varying degrees of nucleus, nucleolus and chromosome irregularities in *A. cepa*. It has been reported in the literature that DNA integrity changes and chromosomal damage occur in *A. cepa* germinating with water samples containing lithium^37^.

**Table 2:** Effects of Li_2_CO_3_ application on CAs frequencies.

	Group I	Group II	Group III	Group IV
SC	0.18 ± 0.28^d^	21.64 ± 1.86^c^	40.80 ± 3.60^b^	79.66 ± 6.16^a^
FRG	0.00 ± 0.00^d^	18.35 ± 1.74^c^	36.47 ± 3.44^b^	70.54 ± 5.98^a^
UDC	0.16 ± 0.24^d^	15.76 ± 1.63^c^	31.54 ± 3.14^b^	62.59 ± 5.52^a^
B	0.00 ± 0.00^d^	11.78 ± 1.46^c^	26.90 ± 2.85^b^	50.80 ± 5.11^a^
VC	0.00 ± 0.00^d^	9.23 ± 1.30^c^	20.36 ± 2.36^b^	41.57 ± 4.66^a^
RP	0.00 ± 0.00^d^	7.60 ± 1.16^c^	16.78 ± 1.94^b^	30.50 ± 3.88^a^
IM	0.00 ± 0.00^d^	5.74 ± 0.85^c^	11.76 ± 1.46^b^	20.55 ± 2.92^a^
MA	0.00 ± 0.00^d^	4.12 ± 0.71^c^	8.15 ± 1.20^b^	15.48 ± 1.84^a^

CAs were calculated by analyzing 1.000 cells in each group. Data with different letters^(a–d)^ in the same line are statically significant at *P* < 0.05.

*SC* sticky chromosome, *FRG* fragment, *UDC* unequal distribution of chromatin, *B* bridge, *VC* vagrant chromosome, *RP* reverse polarization, *IM* irregular mitosis, *MA* multipolar anaphase.

### DNA fragmentation

**Figure 6:**
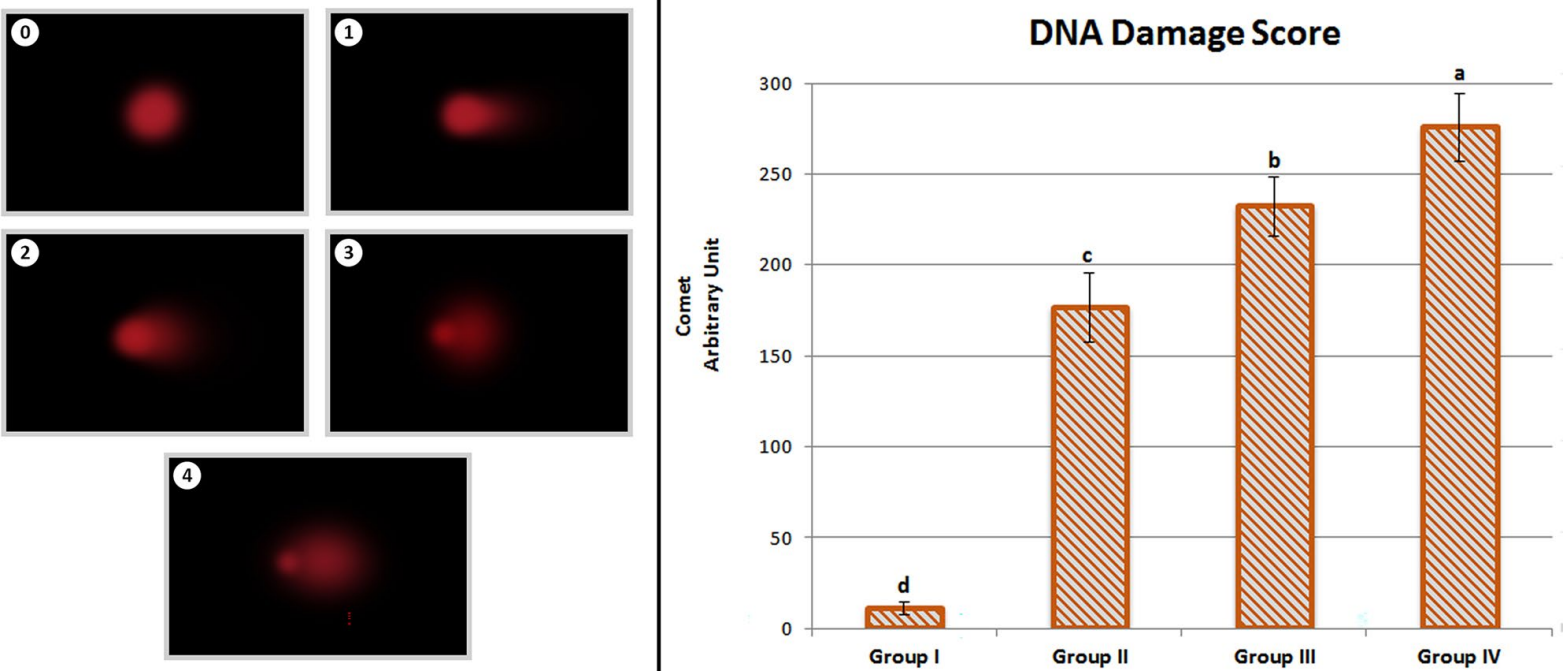
The effect of Li_2_CO_3_ treatment on *A. cepa* root tip cell nucleus (0: no damage, 1: low damage, 2: moderate damage, 3: high damage, 4: extreme damage. A total of 1.000 cells were examined for DNA damage in each group. Data with different letters^(a-d)^ in the columns are statically significant at *P* < 0.05.

DNA strand breaks due to Li_2_CO_3_ application in the nucleus of *A. cepa* root tip cells were evaluated by comet assay. Figure 6 demonstrates the effects of Li_2_CO_3_ treatment on DNA fragmentation in *A. cepa.* The obtained comet assay results showed that Li_2_CO_3_ application caused DNA strand breaks. While the average DNA damage score was 10.83 ± 3.48 in Group I (control), a sharp increase occurred in Group II, which was administered 25 mg/L Li_2_CO_3_, and the average DNA damage score was 176.50 ± 19.35. In Group III, where the Li_2_CO_3_ dose increased to 50 mg/L, the DNA damage score increased to 232.17 ± 16.29. The DNA damage score was determined as 275.67 ± 18.84 in Group IV treated with 100 mg/L dose of Li_2_CO_3_. The DNA damage score increased with increasing Li_2_CO_3_ doses, demonstrating that the occurrence of DNA fragmentation increased as Li_2_CO_3_ doses increased. Our findings are also confirmed by the results of other studies. Although there is no study in the literature investigating DNA fragmentation in plants with Comet, it is reported that lithium compounds cause DNA breaks in some cells^40^.

### Potential interactions of Li_2_CO_3_ with DNA molecules

**Figure 7:**
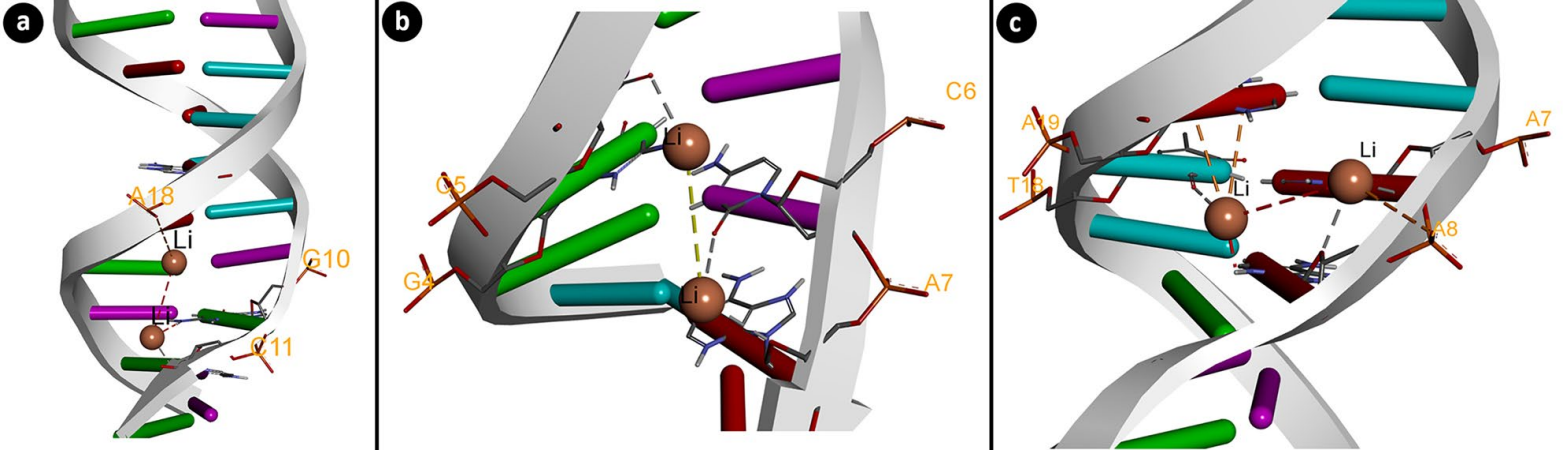
The interactions of lithium with DNA sequences. 1BNA (**a**), 1CP8 (**b**), 195D (**c**).

Lithium binding affinities on DNA were investigated to support the genotoxic mechanism of action of lithium. Table 3 and Fig. 7 show evidence of lithium interactions with DNA sequences. Lithium interacted with B-DNA dodecamer (1BNA) with a binding energy of − 3.24 kcal/mol. Lithium showed molecular interactions with bases G10 and C11 in the A chain and with A18 in the B chain. The interaction of lithium in DNA (1CP8) occurred with a binding energy of − 2.75 kcal/mol. It showed interactions with G4 and C5 bases in the A chain and with C6 and A7 bases in the B chain. Lithium interacted with bases A7 and A8 in the chain A of B-DNA Dodecamer D (195D), with bases T18 and A19 in the chain B with a binding energy of − 3.20 kcal/mol. The findings of molecular docking studies between lithium and various DNA molecules confirmed lithium's capacity to intercalate by engaging with same and distinct strands in DNA. It also shows that lithium may influence DNA structure by binding to areas rich in G-C, C-A, A-A, and T-A nucleotides.

**Table 3:** The binding energy of Li_2_CO_3_ with DNA molecules.

DNA molecule	DNA sequence	Free energy of binding (kcal/mol)	Inhibition constant (Ki) (mM)	Interacting nucleic acids (Chain: nucleotide)
B-DNA Dodecamer (1BNA)	5ʹ-CGCGAATTCGCG-3ʹ	− 3.24	4.22	A:G10
				A:C11
				B:A18
DNA (1CP8)	5ʹ-TTGGCCAA-3ʹ	− 2.75	9.64	A:G4
				A:C5
				B:C6
				B:A7
B-DNA Dodecamer D (195D)	5ʹ-CGCGTTAACGCG-3ʹ	− 3.2	4.51	A:A7
				A:A8
				B:T18
				B:A19

### Li_2_CO_3_ effects on anatomic alterations

**Figure 8:**
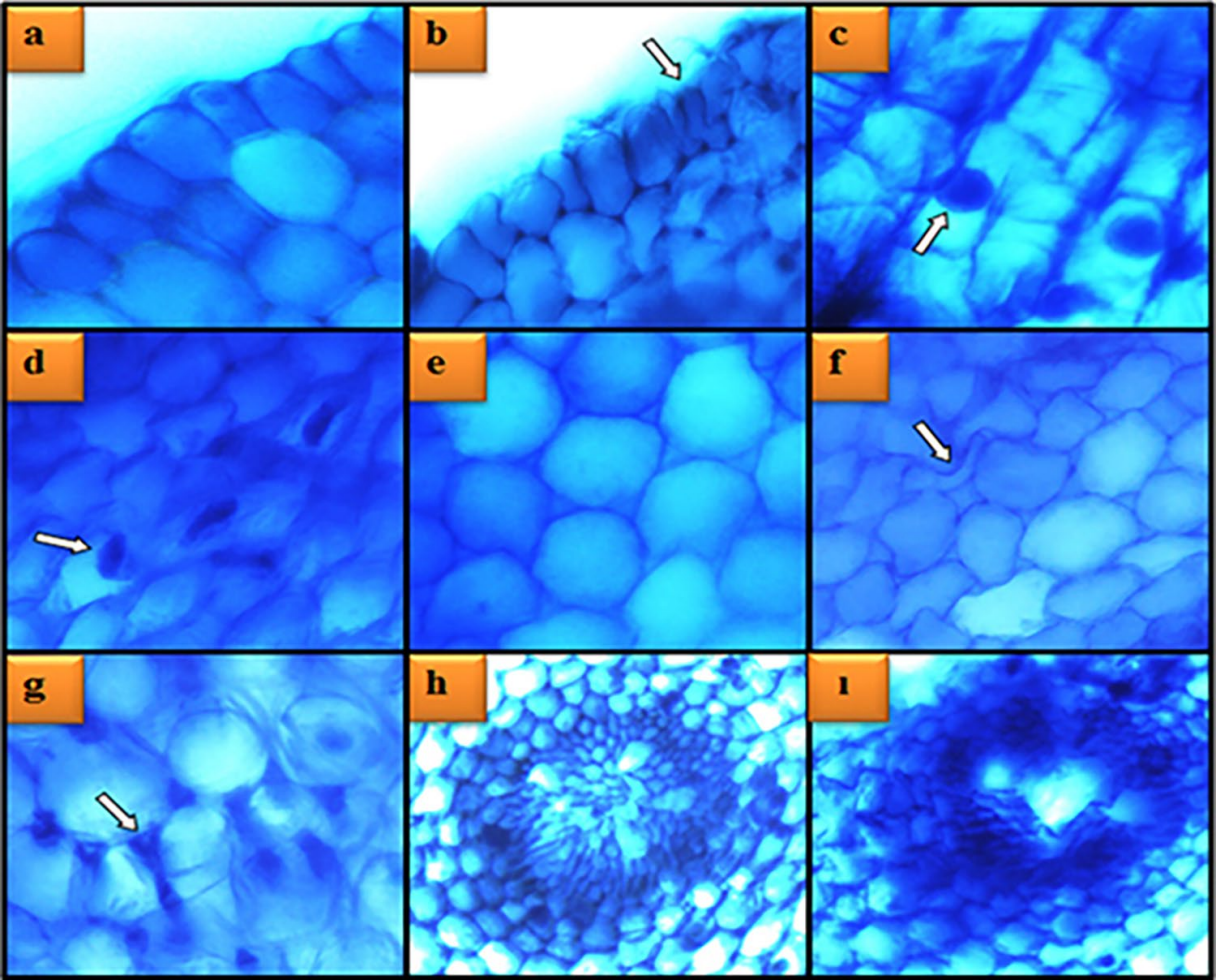
Li_2_CO_3_ induced meristematic cell damages. Epidermis cells in control (**a**), epidermis cell damage (**b**), appearance of cell nucleus in control-*oval* (**c**), flattened cell nucleus (**d**), cortex cells in control (**e**), cortex cell damage (**f**), thickening of the cortex cell wall (**g**), vascular tissue in control (**h**), unclear vascular tissue (**ı**).

The anatomical changes caused by Li_2_CO_3_ application in *A. cepa* are given in Table 4 and Fig. 8. As a result of 100 mg/L Li_2_CO_3_ application, severe epidermis cell damage and cortex cell damage, moderate flattened cell nuclei, thickening of the cortex cell wall and unclear vascular tissue were observed. In 25 mg/L Li_2_CO_3_ application, epidermis cell damage, cortex cell damage, flattened cell nucleus, thickening in the cortex cell wall, and unclear vascular tissue were little, while these parameters were moderate in 50 mg/L Li_2_CO_3_ application. The response of roots to heavy metals is very important because the roots are the main input of metal ions in plants. Plant roots are the first organ to come into contact with lithium in the soil, and excess lithium has been reported to alter the root gravitropic growth of plants^41^. Deformation in epidermis cells and thickening of the cortex cell wall may be a possible defense mechanism of the plant to prevent excess lithium uptake. These results are consistent with the previous results (genotoxicity, oxidative stress) of this study. Considering the increase in MDA induced by Li_2_CO_3_, the structural deformations in the meristematic tissue may be due to oxidative stress-induced damage to the cell membranes. In the literature, there are studies reporting anatomical changes in plants as a result of metal contamination. Yalcin et al.^35^ reported that administration of HgCl_2_ in *A. cepa* caused meristematic cell damage and different anatomical aberrations.

**Table 4:** Effects of Li_2_CO_3_ application on anatomical structure of *A. cepa* meristematic cells.

Groups	ECD	FCN	CCD	TCCW	UCT
Group I	−	−	−	−	−
Group II	**+**	**+**	**+**	**+**	**+**
Group III	**++**	**++**	**++**	**+**	**+**
Group IV	**+++**	**++**	**+++**	**++**	**++**

*ECD* epidermis cell damage, *FCN* flattened cell nucleus, *CCD* cortex cell damage, *TCCW* thickening in the cortex cell wall, *UCT* unclear vascular tissue.

+++: severe damage, ++: moderate damage, +: litle damage, −: no damage.

## Conclusion

In this study, the in vivo toxicity profile of Li_2_CO_3_, which is used for various purposes in many fields, especially in the energy sector, and released to the environment as waste, was investigated using meristematic cells. Li_2_CO_3_ caused a cytotoxic effect by causing a regression in MI rates, and a genotoxic effect by inducing MN and CAs. Genotoxicity mechanism of Li_2_CO_3_, which was determined to cause DNA fragmentation by comet test, was examined by in silico analysis and it was determined that DNA interaction through intercalation was the main cause of genotoxicity. Root elongation and regression in germination indicate that Li_2_CO_3_ negatively affects physiological growth. The possible reason for this effect was related to the antioxidant/oxidant balance, which was disturbed by the abnormalities in MDA and GSH levels and the changes in antioxidant enzyme activities. The contamination levels of lithium to the environment vary between 3.74 and 169.5 mg/kg in soil and 1.58–1700 µg/L in aquatic environments^42^. Industrial contaminations can cause these levels to increase. In this study, it was determined that lithium doses of 25–100 mg/L caused toxic effects in *A. cepa*. It is clear that the use of lithium-containing wetlands in agricultural applications would be dangerous due to the toxic effects on eukaryotic organisms such as *A. cepa.* The toxic effects of Li_2_CO_3_ in *A. cepa*, a eukaryotic and bio-indicator organism can be used as a preliminary assessment for effects in other eukaryotic organisms. The high compatibility of *Allium* test results with other toxicity tests and the fact that it has an oxidase system indicates that the toxic effects determined by this test may also occur in other living things, even mammals. Considering that it is an important environmental contaminant, the detected toxic effects of Li_2_CO_3_, are quite thought-provoking. The rapid spread of technology and consumption-based society increases the interest in electronic products day by day, and a huge amount of e-waste is generated every minute. Unless regular recycling of e-waste is ensured, it also complicates the supply of raw materials used in electronic devices. Extracting the lithium called as clean energy from its ore and converting it into a commercially usable form such as Li_2_CO_3_ or lithium hydroxide (LiOH) accelerates the contamination of the environment. Serious environmental problems occur as a result of both non-recyclable e-waste and the acceleration in lithium mining. Lithium compounds, which reach many organisms through the food chain, also exhibit various toxic effects. For this reason, recycling of lithium-containing e-waste will protect living beings, the environment, natural resources, save energy and prevent fertile lands from being filled with waste.

## Data Availability

The datasets used and/or analyzed during the current study are available from the corresponding author on reasonable request.
